# Filoviruses in Bats: Current Knowledge and Future Directions

**DOI:** 10.3390/v6041759

**Published:** 2014-04-17

**Authors:** Kevin J. Olival, David T. S. Hayman

**Affiliations:** 1 EcoHealth Alliance, 460 W. 34th Street, New York, NY 10001, USA; 2 Department of Biology, Colorado State University, Fort Collins, CO 80523, USA; E-Mail: davidtshayman@gmail.com; 3 Department of Biology, University of Florida, Gainesville, FL 32611, USA; 4 Molecular Epidemiology and Public Health Laboratory, Hopkirk Research Institute, Massey University, Private Bag 11 222, Palmerston North 4442, New Zealand

**Keywords:** bats, Chiroptera, disease ecology, emerging infectious diseases, Ebola, Filovirus, Lloviu, Marburg, Ravn, review

## Abstract

Filoviruses, including *Ebolavirus* and *Marburgvirus*, pose significant threats to public health and species conservation by causing hemorrhagic fever outbreaks with high mortality rates. Since the first outbreak in 1967, their origins, natural history, and ecology remained elusive until recent studies linked them through molecular, serological, and virological studies to bats. We review the ecology, epidemiology, and natural history of these systems, drawing on examples from other bat-borne zoonoses, and highlight key areas for future research. We compare and contrast results from ecological and virological studies of bats and filoviruses with those of other systems. We also highlight how advanced methods, such as more recent serological assays, can be interlinked with flexible statistical methods and experimental studies to inform the field studies necessary to understand filovirus persistence in wildlife populations and cross-species transmission leading to outbreaks. We highlight the need for a more unified, global surveillance strategy for filoviruses in wildlife, and advocate for more integrated, multi-disciplinary approaches to understand dynamics in bat populations to ultimately mitigate or prevent potentially devastating disease outbreaks.

## 1. Introduction and Background

Filoviruses, including Ebola and Marburg viruses, are recognized as a significant threat to public health and conservation as they cause periodic human and non-human primate outbreaks with high mortality rates. Since 1967 when *Marburgvirus* first emerged in humans, their importance as lethal pathogens causing hemorrhagic fever has been appreciated, but their origins, natural history, and ecology remained elusive for decades. In 2005, the first direct evidence from field studies that bats were reservoir hosts for *Ebolavirus* was reported [1], and research has since been growing to understand the role that bats play in the maintenance, transmission, and evolution of filoviruses. There are a number of excellent reviews on the history of filoviruses, their virology, molecular biology, and vaccine development [2,3,4], including a special volume published in this journal “Advances in Filovirus Research 2012” [5]. We do not wish to replicate those previous reviews here and those subjects are not the focus of our paper. Thus, we only briefly review key aspects of filovirus biology before focusing our review on the issue of filoviruses in bats, with a focus on understanding the ecology, epidemiology, and natural history of this system. Through extensive review of the published literature and by drawing examples from research on other bat-borne zoonoses, we will specifically examine the current state of knowledge regarding Marburgviruses and Ebolaviruses in bats and highlight key areas for future research to better understand these associations. 

### 1.1. Basic Virology

The *Filoviridae* family in the order Mononegavirales is separated from other Mononegavirales on the basis of morphological, physiochemical, and biological features [6,7] and more latterly genomic analyses [8]. Filoviruses are non-segmented, negative-strand RNA viruses. The viruses are filamentous (Filo- derived from the Latin *filum* or thread) enveloped particles of variable length. The filovirus genomes are typically approximately 19 kb in length [6,9]. The proteins expressed by the filoviruses are: nucleoprotein (NP), glycoprotein (GP), RNA-dependent RNA polymerase (L), and four structural proteins: VP24, VP30, VP35, and VP40 [9,10]. *Ebolavirus* is able to express a truncated soluble glycoprotein (sGP) through RNA editing. The ribonucleoprotein is derived from the RNA genome, NP, VP30, VP35, and L protein, though *Marburgvirus* is reported to be able replicate in the absence of VP30. The VP35 protein is known to block interferon induction in both Marburg and Ebola viruses [11], and the discovery of the open reading frame for this protein integrated into bat genomes is an area for future research exploration to better understand host-virus interactions and immunity [12]. The two proteins VP40 and VP24 form the internal viral membranes and the surface of the viral membranes are spiked with GP trimers. The trimers are formed from GP1 and GP2, which are cleaved from the GP precursor. The GP trimers mediate receptor binding and are the target for neutralizing antibodies [13].

### 1.2. Viral Taxonomy and Phylogeny

In this article, we defer to the revised filovirus taxonomy of the 9th report of the International Committee on Taxonomy of Viruses (ICTV) including proposals by Kuhn *et al.* [14,15]. Ebolavirus and Marburgvirus are the two currently recognized genera of the family Filoviridae. Lloviu virus [16] may be classified as a distinct genus, Cuevavirus, and species Lloviu cuevavirus [14]. The two classified genera are divided into increasing numbers of species, as more viruses are discovered. Within the genus Ebolavirus, Zaire ebolavirus, Sudan ebolavirus, Reston ebolavirus, Taï Forest ebolavirus (formerly Côte d’Ivoire ebolavirus), and Bundibugyo ebolavirus are recognized species. Within the genus Marburgvirus there is a single species, Marburgvirus marburgvirus (formerly Lake Victoria marburgvirus), which consists of two very divergent “viruses”: Marburg virus and Ravn virus, approximately 20% divergent at a genetic level [8,14,15,17,18,19]. This is in contrast to the known diversity for Ebolavirus species, with Zaire ebolavirus having only a 2.7% nucleotide difference between sequences, Sudan ebolavirus 5.2%, and Reston ebolavirus 4.5% [8,20]. Despite increasing numbers of viruses being detected, some species are represented by single viral lineage (e.g., Taï Forest ebolavirus by Tai Forest Virus and Lloviu cuevavirus by Lloviu virus). These taxonomic classifications will continue to change as increased surveillance in wildlife hosts and humans and genome sequencing will uncover more divergent lineages within Filoviridae, from new localities and new hosts. While viral taxonomy ultimately relies on formal proposals and expert review by the ICTV [11,12], it will also be important to have flexible and more rapid classification schemes in place to assess the taxonomy of new lineages as our knowledge of filovirus diversity grows [20,21]. 

Phylogenetic techniques, in particular coalescent-based models, have also been used to estimate the ages of filoviruses. Interestingly, common ancestor age estimates have ranged from thousands to millions of years [12,16,22,23], suggesting both novel techniques and increased sample sizes are needed, and that better understanding of filovirus evolution (e.g., purifying selection, integration into host genomes, *etc.*) must be gained before reliable dates can be obtained. For individual species, some models have suggested *Zaire ebolavirus* viruses diverged from a common ancestor very recently [24,25,26,27]. Recent analyses using Bayesian coalescent phylogenetic analyses on 97 whole-genome sequences have been able to estimate nucleotide substitutions/site/year for different viruses (ranging from 0.46 × 10^−4^ for *Sudan ebolavirus* to 8.21 × 10^−4^ for *Reston ebolavirus*) [8]. The analysis by Carroll *et al.* estimates recent common ancestry (approximately 50 years ago) for *Reston ebolavirus* and *Zaire ebolavirus*, and the authors suggest these species may have experienced recent genetic bottlenecks. *Marburg marburgvirus* and *Sudan ebolavirus* species were estimated to have common ancestors less than 1000 years ago (approximately 700 and 850 years ago, respectively), whereas the *Filoviridae* were estimated to share common ancestry 10,000 years ago [8].

### 1.3. Filovirus Outbreaks in Humans—Brief History Including Known Links to Bat Exposure

*Lake Victoria marburgvirus* was the first filovirus discovered in 1967, when laboratory workers in Marburg, Germany and Belgrade, Yugoslavia (now Republic of Serbia) were exposed to the virus after contact with infected, imported green monkeys (Chlorocebus spp.). Subsequently, a number of small human outbreaks of Marburgvirus (both Marburg virus and Ravn virus) occurred sporadically between 1975–1997, some of which had some link to bat caves [11,28]. The two largest outbreaks of Marburg virus occurred in the Democratic Republic of Congo (DRC) 1998–2000 where 128/154 infected people died; and in Angola in 2004–2005 where 227/252 patients succumbed to the virus [11]. The DRC outbreak was linked to gold mining in Goroumbwa cave [29], and origins of the Angola outbreak are not certain. Three small outbreaks occurred in Uganda between 2007–2008, one associated with gold mining from Kitaka cave, and two single human cases were Western tourists visiting Python Cave in Uganda while on vacation [28]. Both Kitaka and Python cave are known to harbor large bat populations, and have been sites for follow up studies on Marburg ecology [19,28]. 

The history of Ebolavirus outbreaks in Africa have also been previously reviewed including an excellent summary of outbreaks up until 2005 [30]. Briefly, as described in that review, in 1976 two outbreaks occurred around the same period—one in Eastern Sudan and one in Eastern Zaire—resulting in 53% and 89% mortality and the first discoveries of Sudan and Zaire ebolaviruses, respectively. Subsequently there was one human Ebolavirus case in 1977 in DRC, and a cluster of 34 cases in E. Sudan in 1979. No Ebolavirus outbreaks occurred again until 1994, when there were a series of outbreaks between 1994–1997 and more again between 2000–2004 [30]. There has only been a single, non-fatal case of Taï Forest ebolavirus in humans, a veterinarian who was infected after performing a necropsy on a chimpanzee in 1994 [31]. Bundibugyo ebolavirus was discovered after human cases of hemorrhagic fever in late 2007 in Western Uganda, but the links to an animal reservoir are not clear [32]. A large Ebolavirus outbreak occurred in DRC in 2007 (186 deaths out of 260 cases, 71.5% mortality), and the initial human “index case” was later speculated to have been linked to purchasing freshly killed fruit bats for consumption [33]. Most recently in 2012, there were four distinct outbreaks in Uganda and DRC, one caused by Marburgvirus that was discovered to be nearly genetically identical to sequences collected from bats a few years prior [34]. Currently, in March 2014, there is an ongoing outbreak of *Ebolavirus* in Guinea. At the time of writing, the WHO reported 103 cases or suspected cases with 66 deaths. Polymerase (L) gene sequence analysis suggests that this outbreak is caused by *Zaire ebolavirus*, which is the first time that this virus has been detected in W. Africa [35].

Reston ebolavirus was first discovered in 1989 from laboratory macaques exported from the Philippines to the USA [36,37]. Subsequent detections of the same virus were made in primates in 1992 and 1996 [38], and Reston ebolavirus was found to be circulating in pigs in the Philippines in 2008 [39]. A small percentage of people (1% of 458 exposed individuals) from the 1989 and 1996 events were found to have IgG antibodies to Reston ebolavirus, but were asymptomatic [38]. Reston ebolavirus infection in humans is rare and not known to cause any human disease.

**Figure 1:**
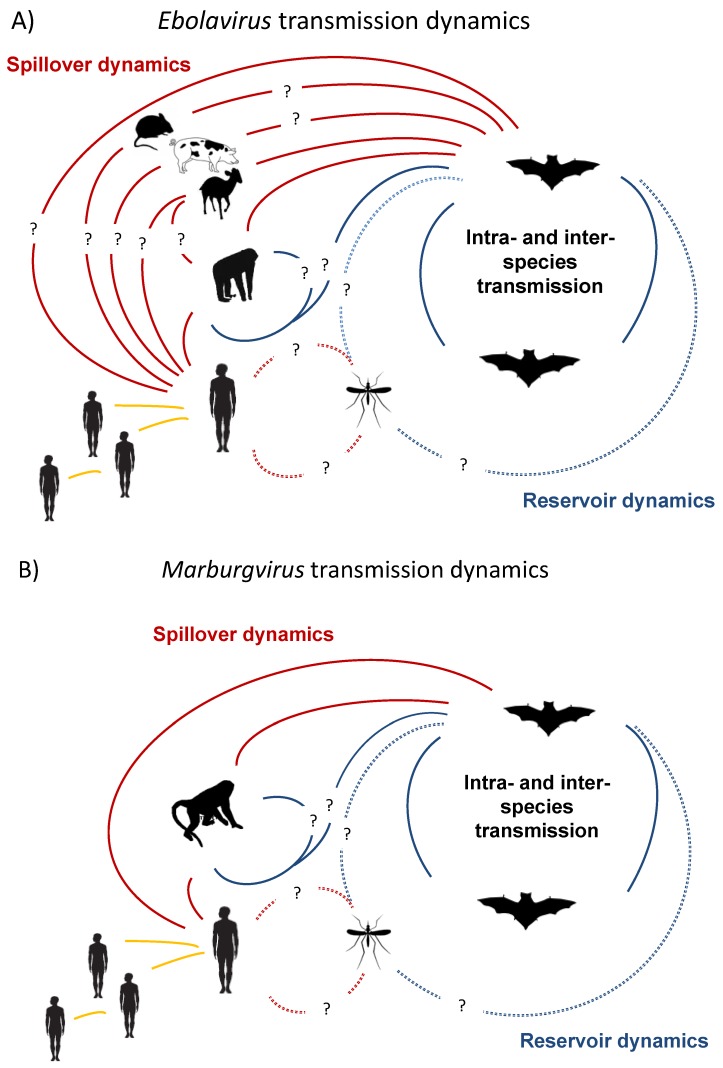
(**A**) The multiple transmission pathways are shown for Ebolavirus genera viruses. The role of vectors is unlikely, but not known (dashed line). Those pathways with epidemiological uncertainty are shown with question marks. Potential reservoir dynamics are shown in blue, spillover epidemics in small mammals (Africa), pigs (Reston ebolavirus only), duikers (Africa), primates and humans shown in red and ongoing human transmission in orange; (**B**) The multiple transmission pathways are shown for Marburgvirus genera viruses. The role of vectors is unlikely, but not known (dashed line). Those with epidemiological uncertainty are shown with question marks. Potential reservoir dynamics are shown in blue, spillover epidemics in primates and humans shown in red and ongoing human transmission in orange.

As noted by others, one interesting feature of filovirus epidemics is that genetic analyses show epidemics can happen as a result of single introduction events into human populations with subsequent human-to-human transmission, or as a result of multiple introductions with less human-to-human transmission (Figure 1), but higher genetic diversity [8,17,40]. Thus, rapid genetic characterization of human and non-human primate outbreaks will continue to be critical in order to better understand the zoonotic and epidemiological origins of filovirus outbreaks [32,34]. Given that molecular tools and high-throughput sequencing (HTS) continue to get cheaper and more efficient, the time from outbreak to full viral genome sequence ready for analysis will continue to decrease and mostly likely be limited by infrastructure for cold-chain and transport of specimens. 

## 2. Natural Reservoirs

### 2.1. Investigations to Find the Natural Reservoir—Elusive for Decades and Ongoing

While there is no consensus on how to unambiguously define an infection “reservoir”, a number of criteria can be applied to identify potential animal reservoirs during epidemiological investigations, and to generally classify when a host species may act as a “reservoir” *vs.* an “accidental host”, see Box 14.1 “What Is a Natural Reservoir for a Pathogen?” in [41]. The natural reservoir for *Marburg-* and *Ebolavirus* remained elusive for decades. Very diverse taxa have been suggested as potential reservoirs for filoviruses over the years, including bats, rodents, arthropods, and plants [42,43,44,45,46]. In a massive field investigation to find the natural reservoir following the 1995 Kikwit, DRC outbreak over 3000 animals were collected primarily from forest areas near the home of the index case, but no evidence of *Ebolavirus* was found [44]. The sampling included 78 mammal species, 51 bird species, and 22 reptiles and amphibians species were collected, and 18 species and approximately 1/5 of all the animals collected were bats. However sample sizes per species were low, with only 4 bat species having greater than 20 individuals collected [44]. Swanepoel *et al.* demonstrated that plants, reptiles, invertebrates and some vertebrates were unlikely reservoirs, because experimentally they were refractory to infections [18]. However the bats they tested (see below) were able to survive infection, support replication, and mount an adaptive immune response. Despite years of investigations, it took nearly forty years from the discovery of *Marburgvirus* in the late 1960s to identify fruit bats as (at least one of) the primary natural reservoir for this virus.

### 2.2. Role of Primates—Potential Reservoirs or Dead-End Hosts?

Primates are known to have a role in filovirus, ion, as the first known human cases were linked to exposure to lab primates in Europe in 1967. Viruses in the genera *Ebolavirus* and *Marburgvirus* have been isolated from infected primates [27,47,48,49], however the role of primates in the natural ecology of filoviruses is still poorly understood and their role as part of a reservoir complex is unknown (Figure 1). Human disease is frequently linked to contact with infected primate carcasses, though direct contact with other infected hosts is reported [18,19,33,50,51] (Figure 1). It is uncertain whether there is primate-to-primate transmission, or if primates are “dead-end” hosts and R_0_ (the number of infections one infected individual causes on average over the duration of the infectious period in a naïve population) is always close to 0. However, it is noticeable that primates, especially great apes, appear to have been severely affected by Ebola (*Zaire ebolavirus*) and populations of western lowland gorillas (*Gorilla gorilla gorilla*) and common chimpanzees (*Pan troglodytes*) have declined by approximately 80% in parts Central Africa and these declines are linked (chronologically and through a small number of molecular studies) to *Ebolavirus* [50,52,53]. Following a human *Ebolavirus* outbreak in Gabon and Congo over a five month period 130/143 gorillas disappeared, with 10/12 gorillas and 3/3 common chimpanzees testing positive to *Ebolavirus* by PCR, antigen capture or immunohistochemical staining post-mortem [52]. These observations suggest that even if R_0_ is less than 1, ape-to-ape transmission may be prolonged enough to cause significant epidemics. Given the many years these ape populations will take to recover after these mass mortality events [53] it suggests that African apes are unlikely to be able act as sole reservoirs for infection. 

In Asia, *Reston ebolavirus* has been isolated from captive primates (*Macaca fascicularis*) in the Philippines (131/1051 were antigen positive) [36,37]. Nidom *et al.* reported anti-*Ebolavirus* antibodies in orangutans (*Pongo pygmaeus*), however, there was substantial variation in titers in orangutans and the study lacked both positive and negative controls [54] that are essential standards required to interpret serological findings [55]. These antibody findings in otherwise healthy orangutans could mean that the filovirus circulating in Asia is less virulent in apes or that orangutans are more resistant to disease (but not infection). Either of which might lead to them being able to act as hosts for filoviruses. Although, like *Zaire ebolavirus*, Reston virus has caused disease and killed primates [36,49,56,57,58,59,60], so if there is an intermediate or novel filovirus circulating in Asian apes it would likely need to be much less pathogenic and cause less disease in apes to persist within these populations. Moreover, a key issue is having a susceptible pool of hosts large enough for pathogens to persist within, which would likely make low density solitary apes, such as orangutans, unlikely reservoirs for acute immunizing infections [61], though they could form part of a complex of multiple species forming a reservoir [62]. Recent evidence for *Ebolavirus* infection in Asian fruit bats species could potentially support the idea that multiple hosts may be involved [63,64]. What is clear is that in Africa apes are susceptible to *Ebolavirus* and may suffer severe disease [52,65,66]. The susceptibility of African apes is both a problem for human health when human–ape contact occurs, as well as a major conservation concern for already threatened species. 

### 2.3. Evidence of Bats as Key Reservoirs—Ebola Viruses and Marburg in Africa

The evidence for bats as reservoirs of ebolaviruses comes from numerous epidemiological and ecological studies. We summarize the known bat host species, methods of detection, and key references for each filovirus species with available data in Table 1. Prior to the detection of Ebolavirus RNA from healthy bats in the field, there were several reasons epidemiologists thought bats may be a reservoirs for ebolavirus. Index cases during Marburgvirus epidemics in Kenya [51,67] gave researchers an epidemiological link between bats and filoviruses when multiple transmission events occurred in mines [68,69,70]. Ecological niche models were used to provide regional perspective on the geographic and ecological distributions of Ebolavirus and Marburgvirus and suggested that various bat, mouse, rat, dormice, and shrew species may be sources of the infection as their distributions overlapped those of all four (then known) African filoviruses [71,72,73]. Other virological studies also suggested small mammals, comprising rodents and shrews, might be reservoirs [74]. Arthropod vectors were also considered, but viral replication in arthropod cell lines was unsuccessful [9,45].

**Figure 2:**
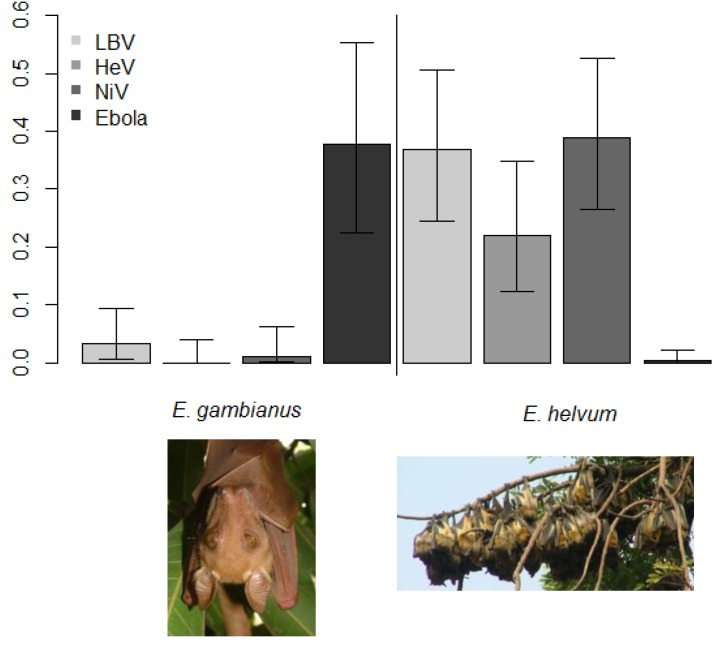
Differing antibody prevalence (as a proportion) from cross-sectional studies of two bat species from Ghana, West Africa. *Epomophorus gambianus* (**left**, Gambian epauletted fruit bat) roosts in low density, is non-migratory and has a high seroprevalence of anti-*Ebolavirus* antibodies. *Eidolon helvum* (**right**, Straw-colored fruit bat) roosts in high density, is migratory and has a low seroprevalence of anti-Ebolavirus antibodies, but high seroprevalence of antibodies against other RNA viruses. The viruses are: Lagos bat virus (LBV), Hendra virus (HeV), Nipah virus (NiV), and *Ebolavirus* (Ebola). Results are adapted from [75,76,82].

To test some of these hypotheses, a wide range of hosts were infected with ebolavirus experimentally in 1996, and bats stood out because they got infected, replicated virus, and survived infection [18]. Finally, in 2005 Leroy *et al.* managed to detect anti-*Ebolavirus* antibodies and *Ebolavirus* RNA in three fruit bat species: *Hypsignathus monstrosus* (24%, 4/17), *Epomops franqueti* (7%, 8/117) and *Myonycteris torquata* (7%, 4/58) after sampling 1,030 animals, including 679 bats, 222 birds and 129 small terrestrial vertebrates [1]. Viral nucleotide sequences were detected in liver and spleen samples (but not other tissues) from *H. monstrosus* (19%, 4/21), *E. franqueti* (4%, 5/117) and *M. torquata* (3%, 4/141). Subsequently anti-*Ebolavirus* antibodies have been detected in numerous other bat species in Africa (Table 1), including high seroprevalences in *E. franqueti* (37%, 10/27), *Epomophorus gambianus* (38%, 14/37, Figure 2), *H. monstrosus* (44%, 7/16), and *Nanonycteris veldkampii* (25%, 1/4) species [75], but notably not in another common fruit bat species [76] in West Africa (Figure 2). Compelling evidence that *Rousettus aegyptiacus* was a key reservoir for *Marburgvirus* came from several studies; and is still the only filovirus to have been isolated from bats [28,30].

**Table 1:** Bat species found filovirus positive by serology or PCR. Bat species listed here for each virus were used to generate the geographic range maps in Figure 3. There are no currently known bat hosts for *Bundibugyo*, *Sudan*, or *Tai Forest ebolaviruses*. PCR = polymerase chain reaction; HTS = high-throughput sequencing. Species synonyms for *Myotis pilosus* and *Tadarida condylura* are used but original host name is retained from original publication.

Virus	Bat Species	Detection Method	References
***Marburgvirus***	*Epomops franqueti*	Antibodies	[77]
	*Hypsignathus monstrosus*	Antibodies	[77]
	*Miniopterus inflatus*	Antibodies; PCR	[18,77]
	*Rhinolophus eloquens*	Antibodies; PCR	[18]
	*Rousettus aegyptiacus*	Antibodies; PCR; Viral Isolation	[18,19,28,77,78,79]
**Lloviu virus**	*Miniopterus schreibersii*	PCR; HTS	[16]
**Reston ebolavirus**	*Cynopterus sphinx*	Antibodies	[80]
	*Hipposideros pomona*	Antibodies	[80]
	*Miniopterus schreibersii*	Antibodies	[80]
	*Myotis pilosus (=Myotis ricketti)*	Antibodies	[80]
	*Pipistrellus pipistrellus*	Antibodies	[80]
	*Rousettus amplexicaudatus*	Antibodies	[64]
	*Rousettus leschenaultii*	Antibodies	[63,80]
**Zaire ebolavirus**	*Eidolon helvum*	Antibodies	[76]
	*Epomops franqueti*	Antibodies; PCR	[30,75,77,81]
	*Epomophorus gambianus*	Antibodies	[75]
	*Hypsignathus monstrosus*	Antibodies; PCR	[30,75,77,81]
	*Micropteropus pusillus*	Antibodies	[77]
	*Tadarida condylura (=Mops condylurus)*	Antibodies	[77]
	*Myonycteris torquata*	Antibodies; PCR	[30,77,81]
	*Rousettus aegyptiacus*	Antibodies	[77]
	*Rousettus leschenaultii*	Antibodies	[63]

### 2.4. Evidence of Filoviruses from Bats in Asia and Europe

**Figure 3:**
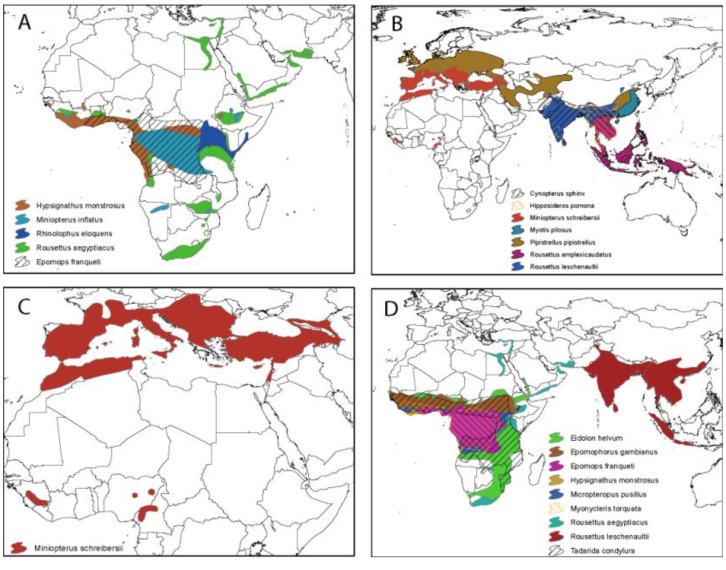
Geographic range for potential bat host species for (**A**) *Marburgvirus marburgvirus*; (**B**) *Reston ebolavirus*; (**C**) *Lloviu virus*; and (**D**) *Zaire ebolavirus*.

In just the past few years, antibody reactive with *Reston ebolavirus* and *Zaire ebolavirus* antigen have been detected in bats from the Philippines, China, Bangladesh, and orangutans from Indonesia (as previously mentioned). Though not conclusive evidence of the presence of these infections, the presence of these or related viruses are not entirely surprising considering the recent discoveries of *Marburgvirus* and *Ebolavirus* from congeneric species (*Rousettus spp.*) in Africa, and considering the large and overlapping geographic ranges for many of these bat species (Figure 3). *Rousettus amplexicaudatus* bats in the Philippines were found seropositive for *Reston ebolavirus* and implicating as the potential reservoir host for this virus in Asia [64]. Additional efforts to identify more solid evidence for *Reston ebolavirus* bat reservoirs in the Philippines and to understand the ecology of bats in this region are underway [83]. In Bangladesh, Olival *et al.* found serological evidence to both *Reston* and *Zaire ebolavirus* in *Rousettus leschenautii* [63]. This was the first evidence for a filovirus infecting wildlife in mainland Asia and suggested that an as-of-yet identified virus, perhaps genetically intermediate between *Reston* and *Zaire ebolavirus*, may be circulating in bat populations there. This Bangladesh bat species was also of particular interest because, along with several other frugivorous bat species in the region, it has close contact with humans and a potential transmission interface through a shared food resource (date palm sap) [63,84]. Yuan *et al.* similarly found *R. leschenautii* to be seropositive for *Reston* and *Zaire ebolavirus* antibodies in China, along with several other insectivorous bat species (Table 1) [80]. 

In 2002, widespread die-offs of Schreiber’s Bent-winged bats (*Miniopterus schreibersii*, Family Vespertilionidae) in the Iberian Peninsula (France, Spain, and Portugal) prompted a wildlife disease investigation. Tissue microscopy from bats collected from a cave in Northern Spain (Cueva del Lloviu) suggested that the bats died from viral pneumonia, and subsequent pathogen screening found that individuals were infected with a novel filovirus, named Lloviu virus [16]. This is the only described filovirus not known to infect humans. This finding was also highly significant as it was the first discovery of an *Ebolavirus* outside of Africa or Asia, and although causation was never proved, it has been speculated that mortality was from Lloviu virus infection. 

### 2.5. Experimental Research Supporting Bats as Reservoirs

Experimental studies supporting the role of bats as reservoirs are few, but two key studies have investigated the capacity for bats to become infected with filoviruses and to survive infection. As mentioned above, Swanepoel *et al.* showed that Zaire ebolavirus could replicate and lead to seroconversion without disease in three species of bats infected (*Tadarida condylura*, *T. pumila*, and *Epomophorus wahlbergi*) and that virus could be isolated from feces [45]. Using captive bred *R. aegyptiacus* bats of known serological and infection status Paweska *et al.* demonstrated that viremia could be induced and Marburg virus detected in multiple tissues 2 to 9 days post infection [85]. Following viremia, IgG antibody could be detected 9 to 21 days post infection. Marburg virus could also be detected in numerous tissues, including lung, intestines, kidney, bladder, salivary glands, and female reproductive tract. None of the bats showed clinical symptoms, nor was gross pathology seen. However, it is worth noting that these studies in *R. aegyptiacus* could not induce infection following oral or intra-nasal inoculation (the above results were following intra-dermal or intra-peritoneal inoculation), nor could virus be isolated from secretions. Similarly, the study by Swanepoel *et al.* inoculated *Tadarida* spp. bats by sub-cutaneous injection; however, fecal shedding was observed in one individual. Thus, while these results are consistent with *R. aegyptiacus* being a reservoir host, they do not shed light on the potential mechanisms for bat-to-bat transmission [85]. Additional experiments underway using a captive *Rousettus* colony housed at CDC Atlanta will likely shed more light on some of these unresolved issues [86]. Lastly, Albarino and colleagues point out that the virus used by Paweska *et al.* was passaged almost 40 times in primate Vero cells prior to infecting bats [87], and it is not known how this may affect the infectivity or virulence of this virus. Reverse genetics can now be used to reconstruct “wild type” *Marburgvirus* strains from genome sequences obtained directly from bats, even in the absence of a viral isolate, and may be a useful tool more relevant than using human or vero-adapted viruses to understand viral dynamics in bats [87].

The recent establishment of bat cell lines [88], including those of the most likely primary reservoir host for Marburgviruses, *Rousettus aegyptiacus* [89], has been invaluable to further unravel the molecular mechanisms of filovirus cell entry and host range in bats. A recent study expressing filovirus envelope GPs on the surface of vesicular stomatitis virus suggest that *Lloviu virus* GP allows viral entry into bat cells more easily than other filoviruses, and thus may be an exceptionally bat-adapted virus [90]. This finding of evidence for adaptation suggests that the bat mortality that prompted the discovery of Lloviu virus may be less likely due to this highly adapted virus, although lyssaviruses are a prime example of host-adapted viruses that remain highly virulent to bat hosts [91]. Additional investigations of host range *in vitro* also using vesicular stomatitis virus expressing GP surface protein, found that *Marburgvirus* was able to infect 6 different bat cell lines from 4 divergent bat genera (*Eidolon*, *Rhinolophus*, *Carollia*, *Tadarida*) [92].

## 3. Filovirus Dynamics and Ecology in Bats—What We Know and Don’t Know

### 3.1. Lessons to Learn from Other Bat Zoonoses

Overall, filovirus ecology remains a neglected area of research, which is understandable as potential reservoirs are still being discovered and for many years remained elusive. Understanding zoonotic disease emergence and cross-species pathogen transmission require multi-disciplinary, process-based approaches that integrate ecological and evolutionary dynamics [93,94]. Several frameworks have been proposed to improve how ecological studies relating to bats and emerging infectious diseases can be performed [95]. Below we highlight some key areas with existing, but limited, information available regarding filovirus ecology and dynamics in bats, and give examples from other bat zoonoses investigations, e.g., research over the past decade into the ecology of Henipaviruses in Malaysia [96,97,98,99,100,101], which may be able to contribute valuable tools or approaches to filovirus ecology research in these areas.

### 3.2. Seasonality of Infection Dynamics in Bats

The most prominent study to test hypotheses regarding bat-filovirus ecology using field approaches and longitudinal sampling is by Amman *et al.*, who looked at breeding cycles and their relationship to Marburgvirus prevalence [28]. Given that many aspects of bat biology, such as mating, birthing, and migration (e.g., [28,76,102,103,104]) are seasonal, Amman *et al.* were the first to test the hypothesis that birthing might be linked to increases in infection prevalence and ultimately spillover for Marburgvirus in bats. Prevalence of other bat derived viruses, including coronaviruses and rabies, are reported to show seasonal dynamics [105,106,107] and the increase in susceptible hosts and contact rates during the birthing period may drive infection dynamics [108]. Their study of Marburgviruses in *R. aegyptiacus* bats in Python Cave, Uganda discovered 2.5% of the bats were actively infected by PCR (and some yielding *Marburgvirus* isolates) [28]. Their analyses suggested *Marburgvirus* infection occurred in distinct pulses in older juvenile bats (approximately 6 months old), coinciding with twice yearly birthing seasons. The authors also reviewed previous human infections and found that most (83%, 54/65) occurred during this same high prevalence/seasonal birth period. Relatedly, Pourrut *et al.* 2009 found that pregnant females bats were statistically more likely to be seropositive for Ebola virus [77]. As many bats have synchronous mating and birthing [103,104,109,110,111,112] and births increase population size and contact rates, the influx of susceptible juveniles may be a central driver of bat infection dynamics. Recent theoretical studies using stochastic epidemiological models with a seasonal birth pulse suggest increased synchrony of birthing increases the necessary critical community size necessary for infection persistence [113]. Thus, seasonal birthing may decrease the probability of pathogens persisting in a colony, but lead to increased periods of infection prevalence following birthing. Whether this is true of all filoviruses in all locations is unknown and further field studies, integrated with modeling, are necessary to understand the role of host ecology on the persistence and emergence of filoviruses in bats [93,95]. 

Evidence from other bat-infection systems suggests that RNA virus shedding may be linked to host ecology and seasonality. Drexler *et al.* studied a maternal colony of *Myotis myotis* bats for three years and showed that RNA viruses (coronaviruses and astroviruses), but not DNA viruses (adenoviruses) were increasingly detected in greater numbers (by quantitative PCR) during colony formation and after parturition [106]. Wacharapluesadee *et al.* showed that Nipah Virus (NiV) in *Pteropus lylei* bats has seasonal dynamics, but with different dynamics for different strains, with a Bangladesh NiV strain more frequently observed April to June and a Malaysian NiV strain found from December to June [114]. These more complex patterns are also suggested by Plowright *et al.* who modeled the transmission dynamics of Hendra virus (HeV) in Australian Pteropid bats and found that their models fit the available data better when population connectivity and immunity (including waning maternal immunity) interact, suggesting more complex dynamics than a simple increase in susceptible juveniles providing enough young for persistence [99]. 

There remain, however, uncertainties about how strong the effects of seasonal birthing are for other filoviruses, and how much coloniality (as shown by *R. aegyptiacus*) and other factors drive infection dynamics. Further still, it has recently been demonstrated that host population structure may be a useful tool to predict infection presence [115] and this remains to be seen for the potential reservoirs of filoviruses. Interestingly, the sub-Saharan African species, *Eidolon helvum*, has been shown to have a high seroprevalence of antibodies against several RNA viruses, but not filoviruses compared to other species in the same locations [76,82] (Figure 2). Given this species is ecologically similar in some ways to *R. aegyptiacus* (seasonal, synchronous birthing; colonial; frugivorous), it poses the question as to whether the ecological differences prevent filovirus circulation (*E. helvum* is migratory; tree roosting) or if there are underlying genetic host restrictions. 

### 3.3. Viral Shedding and Immunity in Bats

There is little understood about filovirus shedding and persistence in bats, though several key studies [1,45,85] suggest that the within-host infection dynamics are the classical “susceptible—infected—immune[recovered]” (SIR) cycle [108]. Swanepoel *et al.* showed that in experimental infection studies Ebola virus replicated in the three species of bats infected (*Tadarida condylura*, *Tadarida pumila*, and *Epomophorus wahlbergi*) with virus isolated from feces 21 days after infection [45]. The bats also seroconverted, suggesting recovery with an adaptive immune response. Leroy *et al.* showed that anti-*Ebolavirus* IgG-positive animals were not *Ebolavirus* PCR-positive, and *vice versa*, suggesting again that infection occurs and is followed by seroconversion [1]. In Amman *et al.* showed that *R. aegyptiacus* bats were discovered to have Marburg virus PCR-positive lung, kidney, colon and reproductive tissues, which may suggest transmission by oral, urine, fecal, or sexual means [28]. The finding of widespread antibody positive bats (Table 1) suggests that survival following filovirus infection is common among bat species. The most compelling evidence for the long-term survival of free-ranging bats following Ebola virus infection is a study by Hayman *et al.*, in which a seropositive bat was known to be alive 13 months after release with a radio collar [76].

### 3.4. Multi-Host and Multi-Pathogen Dynamics in Bats

Multi-species interactions are critical to understand in order to accurately model viral dynamics in bat populations. To date, there is evidence for filovirus infection in a total of 17 bat species for (Marburgvirus, Zaire ebolavirus, Reston ebolavirus, and Lloviu virus), but no currently known bat hosts for Bundibugyo, Sudan, or Tai Forest ebolavirus (Table 1). Virus has only been detected via PCR and sequencing in 7 (41%) of these potential bat reservoir species, and some serological findings listed in Table 1 are sparse (e.g., only 2/679 Epomops franqueti seropositive for Marburg virus [77]. Multiple bat species could potentially act as reservoirs for Zaire ebolavirus, Reston ebolavirus, and Marburg virus, but only one host species is currently known for Lloviu virus (Table 1). Many of these species have overlapping geographic ranges, and have the potential (at a geographic, not necessarily ecological, scale) to interact and share pathogens (Figure 3). However, while either fragments of virus (PCR) or antibodies were detected in these hosts, their true role as reservoirs *versus* incidental hosts and the relative contribution of each species to interspecific host dynamics is currently unknown. Multiple circulating pathogens can also change within-host and within-population dynamics and could confer cross-species immunity [93]. For example, multiple divergent Marburgvirus strains circulate within a single roost of R. aegyptiacus [19,28]. This poses interesting questions regarding how these pathogens interact, such as is there cross-immunity and do divergent viruses have the same infection dynamics? Though cross-reactivity is shown among ebolaviruses, it is unknown how this translates to immunity within the hosts [116]. Leroy *et al.* demonstrated numerous bats infected (detected by PCR) with similar Zaire ebolavirus species PCR fragments some years apart, but within the species, these short genomic fragments differed between species and collection time [1]. In both cases, multiple hosts and circulating pathogens can complicate our understanding of virus-host interactions and should be considered during study design [93].

### 3.5. Meta Populations and Connectivity

Another key aspect of ecological theory that must be investigated further is the role that meta-population dynamics may play in the ecology and evolution of filoviruses. Amman *et al.* provided evidence of direct movement between different caves for *R. aegyptiacus* and have found that there is genetic similarity between viruses detected in geographically distant locations [28]. They suggest that *R. aegyptiacus* exist as a large meta-population with virus circulation over broad geographic ranges. Population genetic studies using mitochondrial and microsatellite markers have confirmed that a congeneric species, *Rousettus leschenaultia*, is highly vagile and panmictic across large areas (e.g., from India throughout China) [117]. Further investigations to understand host movement and connectivity of potential filovirus reservoirs are warranted.

Several previous studies have investigated the relationship between host population structure and bat viral dynamics. Olival *et al.* showed that *Pteropus vampyrus*, the primary natural reservoir for NiV in mainland Southeast Asia, was highly vagile and panmictic using both host and parasite genetics, and was likely the primary player in NiV transmission and circulation [97,98,118,119]. Plowright *et al.* suggested meta-population dynamics were necessary for HeV persistence in Australian Pteropid bats and they predicted reduced connectivity leads to larger epidemics within bat colonies due to a greater loss of herd immunity in colonies with lower levels of connectivity [99]. More recently, Peel *et al.* have used host panmixia to predict infection dynamics across sub-Saharan Africa and shown similar antibody prevalences against two viruses, Lagos bat virus (a lyssavirus) and an as yet undetermined henipa-like paramyxovirus [115]. This species has been shown to both breed freely enough that there is panmixia [115] and travel between roosts over shorter time spans [104], suggesting movement between colonies within the period short enough for infection to occur and for a bat to become infectious [45]. These meta-population dynamics will be important to consider when designing future ecological studies and modeling bat-filovirus data.

## 4. Future Directions in Bat Filovirus Research

### 4.1. Unexplored Diversity and Geographic Gaps—A More Unified Surveillance Strategy

There are over 1200 bat species globally and only a small fraction (~15%) have been targeted for viral discovery to date [41]. That said, pathogen discovery in bats is becoming a widespread activity globally, and this presents an opportunity for researchers to screen specimens for filoviruses while running other routine assays. Global surveillance programs like CDC’s Global Disease Detection centers, or United States Agency for International Development’s (USAID) Emerging Pandemic Threat Program have established laboratory protocols for screening specimens from a diversity of wild mammal hosts. For example, the USAID PREDICT project uses degenerate PCR primers to screen bats, rodents, and primates across multiple (~10–20) viral families including Filoviruses in 20 countries around the world [94]. Through capacity building in emerging infectious disease “hotspots” globally [120], these efforts have the potential to establish a new baseline for the “unknown” zoonotic pool in wildlife and redraw the biogeographic boundaries of pathogen distribution and host range [94,121]. These global, coordinated efforts may allow us to identify novel viruses that have not yet emerged into human populations and develop prevention strategies to ensure that they do not. Lloviu virus is a good example of this, as it was picked up during wildlife surveillance after a die-off in a bat population [16] and is now part of follow-up studies to better understand its genome [8], molecular biology, and cell entry [90]—in part to be able to predict its potential to spillover and infect humans.

While it is important to survey wildlife showing clinical signs of disease, most viruses are discovered in bats from asymptomatic animals, and a two-pronged approach of screening both healthy and diseased animals is required [122]. Modeling approaches to target bat host species based on life-history traits [123,124] or viral “habitat” suitability using ecological niche models [71,72,73] can both be used to refine the taxonomic and geographic scale of surveillance for novel filoviruses or novel filovirus host species. 

### 4.2. Develop More Sensitive, Non-Invasive Tools for Longitudinal Monitoring of Bat Populations

As part of a more unified filovirus surveillance strategy in bats, it will also be necessary to develop non-invasive sampling protocols *and* better detection methods for viral discovery [121,125]. Following an experimental inoculation, Swanepoel *et al.* demonstrated that *Zaire ebolavirus* could be detected in bat feces, but few studies to date have routinely screened bat excreta by PCR in the field. There are also limited data comparing viral detection from organ specimens with data from excreta collected from the same animals. Developing more sensitive assays to detect antibodies or virus from small quantities of blood [126] or bat excreta [121], respectively, has two potential benefits. First, bats (of which many species are threatened) do not need to be killed to identify potential filovirus reservoirs, or study the distribution and the seasonality of viral shedding or infection. Second, for management interventions, it is most important to understand the routes of viral shedding in bats and the seasonality of this shedding, rather than the presence or absence of a virus in a given animal or tissue type. Thus, there may be more value in detecting a virus in bat feces, urine, or saliva than there would be in bat tissue *if* transmission is occurring indirectly in bat habitat (caves or mines). However, if the risk interface is through bushmeat hunting and direct butchering of bats [33], then understanding prevalence and viral load in tissues and blood would be most relevant. 

**Figure 4:**
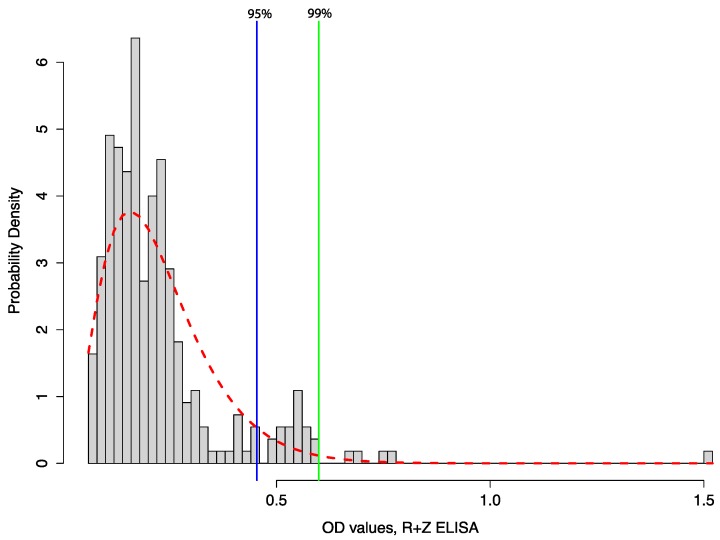
Methodology for determining potentially positive (*i.e.*, cut-off values) for bat individuals using serological data. This figure highlights some of the challenges in interpreting filovirus serology (cut-off values) in bats, and why these data should be examined carefully. Distribution of Optical Density (OD) values from ELISA assay using 1:1 mixture of recombinant nucleoproteins for *Reston* + *Zaire* (R+Z) *ebolavirus* in *Rousettus leschenautlii* fruit bats. Data from [63], using methodology adapted from [77].Cut-off values were determined to be >0.454 for the R+Z ELISA using a maximum likelihood estimator, gamma distribution, and 95% risk of error.Pourrut *et al.* 2009 used an exponential distribution, but the data here are better fitted to a gamma distribution. This approach is less arbitrary than the standard approach of using a value 3× the OD value of negative control, as it uses the distribution of the data itself and a statistical framework to identify potential positive cut-off values. Grey bars = OD values from individual bats for the R+Z ELISA (without positive or negative controls); red line = gamma distribution; blue = 95% confidence of cutoff values; green = 99% confidence. After initial screening, 15 (11%) of 141 *R. leschenaultii*, 6 (8%) of 75 *Cynopterus* spp., and 4 (7%) of 56 *Megaderma lyra* bats were potentially positive at the 95% confidence level. However, only 5 (3.5%) of 141 (95% CI 1.5%–8.0%) of *R. leschenaultii* bats were reported as seropositive after additional testing by ELISAs and Western Blot [63].

There is also a need for better studies of immunological responses in bats [127]. Understanding bat immune responses to filovriruses will help understand the ecology of these viruses within the natural setting because it can be challenging to interpret antibody data in wild species and difficult to use these data to decide whether or not a species is a reservoir (see Figure 4). More specific and sensitive assays, such as Luminex technology [128] and pseudotype assays [129] may help resolve some of these issues. Baker *et al.* demonstrated how accurate quantification of antibody responses using Luminex technology was able to demonstrate the potential effects of pregnancy on henipavirus transmission in a captive study of *Eidolon helvum* that would not have been possible with assays that used dilution series or provide binary responses [130]. These assays still require positive and negative controls, but Peel *et al.* have shown how similar data can be analyzed in the absence of validated gold standard assays from the appropriate species and population (and applied these methods to bat sera) [131]. These approaches, however, remain problematic without better knowledge of the immune response of the bat species to a particular virus. Depending on how cut-off values are determined, some studies can easily overestimate the seroprevalence of a given populations or species. Statistical tools that consider antibodies as noisy populations of antibodies, rather than a binary process, and seek to delineate cut-offs for epidemiological studies, are useful tools for understanding serological assays and have used by several authors including Peel, Pourrut, and Olival [63,77,131] (Figure 4). However, ultimately researchers should aim to understand the dynamic antibody responses in the appropriate species infected with the appropriate virus by an optimal assay before interpreting field data, despite this being difficult in practice. 

### 4.3. Genomics and Viral Fossils

The number of vertebrate genomes available for bioinformatic studies will continue to rise in the next decade as the both the cost and effort needed for sequencing them continue to decline. Advances in HTS can also offer insights into viral evolution, by offering rapid, culture free methods that allow analyses of whole viral genomes (e.g. [8]), as well as characterizing the host genome of potential reservoir species. The recent HTS of two bat genomes offers tantalizing, but preliminary, insights into how bats may be adapted for flight and perhaps have altered innate immune systems that suggest bats may respond to viral infections in a subtly different way to other mammalian hosts studies to date [132]. Understanding host responses to filovirus infection and details of host-viral interactions at a genetic level may improve understanding of field data and enable researchers to develop more nuanced methods of interpreting serological assays, and modeling infection dynamics. 

Several *in silica* studies published in the past few years have used genomic data and identified filovirus genes (endogenous viral elements, EVEs) integrated into the genomes of several mammalian species, including bats [12,23,133,134]. One particularly interesting avenue for future research is whether integration of these viral genes confer some immunological advantage to hosts [134]. Evidence for this is supported by the fact that in some cases long open reading frames for these endogenous viruses have been preserved in host genomes for over thousands of years and that their presence correlates with the absence of disease in host species [134]. With the availability of more data, additional comparative genomic studies that seek to understand the phylogenetic distribution of these endogenous viruses in mammalian hosts may help to inform why some bat species appear to be resistant to infection; but also could be used to identify potential filovirus reservoir hosts that are not yet known [133]. For example, Katzourakis *et al.* found a strong association of endogenous filoviruses elements in both rodents and marsupials—pointing to these groups as potentially important reservoirs, although currently not known to harbor exogenous filoviruses [133]. These *in silica* analyses may be of use to help target which of the ~5000 mammals species to focus efforts for exogenous filovirus discovery and can be part of a more unified strategy for global filovirus surveillance. 

### 4.4. Better Understanding Viral Shedding and Transmission in Bats

While we have a decent understanding of the progression of infection and immunity in individual humans [3], little is known about antibody persistence and viremia in bats. Experimental infections studies in captive bats and long-term monitoring of bat populations in the field using mark-recapture should help to inform this. As previously mentioned, a large number of outbreaks have been directly linked to mining activities or cave exposure [40,51,67,135,136,137]. However, the route of transmission is uncertain—is infection through aerosolized droplets of bat excreta and inhalation, or through some other mechanism? Experimental studies will shed light on these mechanisms and routes of exposure and can be used to guide policy to mitigate spillover. 

While experimental studies with BSL-4 agents such as filoviruses can be challenging, captive studies can be used to understand infection and antibody dynamics in the absence of experimental challenge. Two studies of henipavirus infected or seropositive fruit bats have been undertaken and show the temporal dynamics of antibodies. Though both studies raise many additional questions, they allow researchers to better understand the results of field studies [130,138]. Experimental studies of filoviruses in primates have been useful to describe filovirus infection, including the symptoms, inflammatory response, viral shedding and therapeutic potential of immunoglobulins in primates [139,140]. 

Experimental studies of other bat derived viruses and their non-bat hosts have been used to try to tease apart spillover transmission mechanisms. Horses can be infected with HeV through intranasal infection, suggesting inhalation may be a potential route of infection [141], but epidemiological studies of human filovirus infection suggest more close contact is required for human transmission. Pigs, hypothesized to have been infected with NiV following ingestion of excreta contaminated/partially eaten fruit, have been shown experimentally to be susceptible to infection following ingestion of NiV, with nasopharyngeal shedding [142]. Following the discovery of swine as a potential host for *Reston ebolavirus* [39], pigs have been used as experimental models. The significance of pigs in filovirus transmission has been discussed elsewhere [143,144], however, experimental studies have shown that *Zaire ebolavirus* can be transmitted from pigs to cynomolgus macaques without direct contact [145]. The mechanism(s) of transmission to primates, which are epidemiologically linked to several filovirus outbreaks and are severely affected by infection, remain unknown. Again, these studies are useful for understanding whether transmission to target, novel hosts is possible, but do not necessarily elucidate the mechanisms for transmission of filoviruses between putative reservoir bat hosts or bats and non-bat species. Studies of transmission mechanisms between and from bats to target species, such as pigs and primates, are a priority for experimental studies. The examples from other systems, in particular the henipaviruses HeV and NiV, suggest that similar studies could be used to identify potential transmission pathways (Figure 1). While there are many inherent difficulties with performing such studies for filoviruses, including extensive field situations, BSL-4 level facilities, and ethical issues, these experiments could greatly improve our understanding of filovirus ecology.

### 4.5. Better Understanding Host Ecology and Spillover Potential to Humans

While there is evidence to support specific instances of viral spillover, the epidemiological links between bats, Ebolaviruses, and human and primate infection are not clear. Recent epidemiological surveys following an outbreak reported increased bat activity through bat migration and hunting prior to an outbreak of Ebola virus in DRC [33]. One recent study found a high prevalence (15%) of IgG antibodies to Zaire ebolavirus in human populations in Gabon, and that populations living in forest areas were at a higher risk to being seropositive as compared to human populations in the grassland, savannah, and lake area [146]. Interestingly, no significant differences in seroprevalence were found between populations that hunted or had contact with animals *vs.* those that did not. 

Several authors have speculated that, like that suspected proximate cause for the NiV outbreak in Malaysia [147], bats may drop partially eaten, *Ebolavirus*-contaminated fruits that terrestrial mammals eat and become infected [148]. In Bangladesh video surveillance has shown bats having direct contact to palm sap, an epidemiological link to NiV infection in human [84] and studies have shown NiV can survive on the surface of mango flesh for up to 2 days [149]. The role of fruit tree masting in inter-species interactions and filovirus spillover, e.g., between frugivorous bats, ungulates (duikers), and primates in the forest, is suspected but not known. Similar video studies to those in Bangladesh have shown how apes in Africa share fruit resources [61], but it is currently unknown if partially eaten fruits can lead to infection with filoviruses. Greater use of such surveillance technology may shed further light on transmission pathways in the filovirus-bat systems. 

Models using the SIR structure have been used for human epidemic dynamics [150,151,152] but not for wildlife. Multi-species SIR models [108] could be developed to describe filovirus transmission within bats and between bats and other host species (e.g., gorillas) and could be parameterized using data from field and experimental investigations. These epidemiological studies could be used to answer questions regarding the transmission processes, including if the virus(es) could persist within specific populations or species alone. These models may also be used to highlight which aspects of host and virus biology may be important and require further study, through the use of sensitivity analyses [95]. As we have previously described, population genetic tools can be used to define geographic limits of populations and quantify connectivity between bat populations for each host species known to harbor a given filovirus species. Fine scale gene flow data can be combined with GPS or satellite telemetry and GIS modeling of landuse change—to assess if environmental features (available habitat) spatially correlate with observed breaks in gene flow and population limits. Satellite telemetry studies in the Philippines, as part of multi-disciplinary investigations of viruses have shown non-random foraging and increased roosts compared to previous knowledge [83]. These types of studies can highlight important aspects of host ecology, as well as the impacts and distributions of infected or previously infected individuals [76]. 

Collectively, these ecological studies will be critical to inform disease management options. For example, management options that reduce human–bat contact during seasonal periods of high risk viral shedding, or at key interfaces, will likely be the most effective approaches and can balance both conservation and human health needs [63,95]. The need to better understand the ecology of filoviruses in their natural hosts and factors that facilitate transmission could not be timelier, as an unprecedentedly large human Ebola virus outbreak is currently ravaging Guinea [35]. We advocate for more integrated, multi-disciplinary approaches to understand filovirus dynamics in bat populations, and to mitigate and control these potentially devastating disease outbreaks.
